# Oxidative Status of Goats with Different CSN1S1 Genotypes Fed *ad Libitum* with Fresh and Dry Forages

**DOI:** 10.3390/antiox9030224

**Published:** 2020-03-09

**Authors:** Daniela Giorgio, Adriana Di Trana, Paola Di Gregorio, Andrea Rando, Marcella Avondo, Adriana Bonanno, Bernardo Valenti, Antonino Di Grigoli

**Affiliations:** 1 Scuola di Scienze Agrarie, Forestali, Alimentari e Ambientali (SAFE), University of Basilicata, Viale dell’Ateneo Lucano, 10, 85100 Potenza, Italy; giorgio_daniela@alice.it (D.G.); paola.digregorio@unibas.it (P.D.G.); andrea.rando@unibas.it (A.R.); 2 Dipartimento di Agricoltura, Alimentazione e Ambiente (Di3A), University of Catania, Via Valdisavoia 5, 95123 Catania, Italy; mavondo@unict.it; 3 Dipartimento Scienze Agrarie, Alimentari e Forestali, Università degli Studi di Palermo, Viale delle Scienze, 90128 Palermo, Italy; adriana.bonanno@unipa.it (A.B.); antonino.digrigoli@unipa.it (A.D.G.); 4 Dipartimento di Scienze Agrarie, Alimentari e Ambientali, University of Perugia, Borgo XX Giugno 74, 06121 Perugia, Italy; bernardo.valenti@unipg.it

**Keywords:** goat, diet, fresh forage, CSN1S1 genotype, redox balance, enzymatic antioxidants

## Abstract

Forty late-lactation Girgentana goats were used to study the effect of diets fed ad libitum and αS1-casein (CSN1S1) genotype on redox balance. The goats genotyped at CSN1S1 locus (A/A, A/F) were subjected to four feeding treatments different for percentage inclusion of dry and fresh forage: DAF100 (98% of Dry Alfalfa Forage), DAF65 (65% of Dry Alfalfa Forage), FSF100 (100% of Fresh Sulla Forage) and FSF65 (65% of Fresh Sulla Forage). Blood samples were analyzed for superoxide dismutase (SOD) and glutathione peroxidase (GPX) activity, reactive oxygen metabolites (ROMs), biological antioxidant potential (BAP) and non-esterified fatty acids (NEFA), beta-hydroxybutyrate (BHBA), albumin, glucose and cholesterol contents. The oxidative stress index (OSI) was calculated as percentage ratio of ROMs to BAP. Redox balance was improved by Sulla inclusion, as reflected in the lower OSI values found in FSF100 and FSF65 groups. DAF100 group displayed the highest GPX activity, while other groups exhibited the highest SOD activity. Fresh forage diets increased albumin concentration while no effect of tested factors was noted on glucose, NEFA, BHBA and cholesterol contents. The interaction diet × genotype was significant only for GPX activity. GPX and albumin were negatively correlated and were correlated positively and negatively with ROMs, respectively. Diet rather than genotype affects redox balance in dairy goats and a possible role of forage polyphenol compounds on oxidative status needs to be tested in future studies.

## 1. Introduction

The oxidant/antioxidant balance in livestock animal plays an important role in nutrition, metabolism and health of farm animals, as reviewed elsewhere [1,2]. The impairment of redox balance, due to an excessive production of prooxidants and/or a depletion of antioxidant defense capacity, exposes livestock animals to oxidative stress. The imbalances in oxidative status may impair animal health both directly by peroxidation of lipids and macromolecules and indirectly through modification of important metabolic pathways [3]. To keep reactive oxygen species production at low levels, tissues are equipped with a powerful antioxidant defense system that include endogenous non-enzymatic antioxidants (e.g., albumin, bilirubin, glutathione), enzymatic antioxidant (e.g., superoxide dismutase, catalase, glutathione peroxidase) and exogenous antioxidants (e.g., tocopherols, carotenoids, vitamin C and polyphenols) working in synergy [4].

During their production life, ruminants are normally subjected to many environmental and metabolic challenges responsible for the alteration of oxidative status. In the evaluation of factors predisposing animal to redox imbalance, the drastic metabolic changes and the increasing nutritional demands occurring during the peripartum period has received great research attention. Several studies have reported variable levels of oxidative stress during the transition period in dairy cows and have been recently reviewed [5]. Changes in redox status indicators were also found in a native sheep breed [6,7] and in a synthetic SCP (37.5% Polish sheep, 12.5% prolific breed, Romanowska or Finnsheep, 25% Suffolk, 25% Charollais) line [8] around lambing and during lactation. Studies in Red Syrian goats report a significant decrease in glutathione peroxidase (GPX) activity and albumin concentration during the peripartum, suggesting that animals experienced moderate oxidative stress during this period [9]. Changes in total oxidative and antioxidant status were observed in local goats from early lactation to late lactation period and it was supposed to be associated to a different lipomobilization occurring in adipose tissue along the lactation period [10]. An influence of parity on the occurrence of peripartum redox imbalance was also observed when the metabolic and antioxidative status of Saanen goats of different parity were compared [11].

Animals living at high altitudes are exposed to severe environmental stress associated with very low atmospheric pressure, oxygen tension, temperature, relative humidity and high ultraviolet exposure [12]. Among environmental factors responsible for redox imbalance, thermal stress is currently gaining more attention under the changing climatic scenario [13]. The exposure to elevated ambient temperatures was suggested to be responsible of inducing redox imbalance in livestock animals [14]. Higher erythrocyte oxidants were observed in indigenous sheep reared in summer than in winter [15]. Higher plasma reactive oxygen metabolites (ROMs) concentration observed in mid-lactation goats during summer compared to spring indicates that goats may have experienced moderate oxidative stress during hot season [16].

It is important to note that the oxidative status in ruminants can be influenced by nutrition. It has been observed that increasing the diet starch content in high yielding dairy cows during early lactation contributes to increase the redox status imbalance, possibly due to cellular changes related to oxidative phosphorylation [17]. The administration of high starch diet in lactating sheep had less evident effects on oxidative stress markers, while an imbalance of oxidative status was found when an enrichment of their diet from 2.6% to 5.4% in fat occurred [18]. A moderate effect of feeding regimes (pasture vs. pasture plus supplement vs. hay plus supplement) and nutritional level of the diet (80% vs. 140% energy requirements) on ROMs content was reported in dairy goats [1,16]. In dairy cows, a short grazing time (5 h/day) on barley grass was not able to increase the antioxidant protection, which was unchanged in comparison with the level exhibited by confined animals [19]. More recently, it has been demonstrated that both under-and overfeeding cause changes in the antioxidant enzymes activities, antioxidant capacity and oxidative stress biomarkers in sheep and goat blood plasma [20].

An increasing strategy in the control of redox homeostasis in ruminants is the dietary administration of nutraceuticals with antioxidant properties [21,22]. Dietary polyphenols have been proposed to exert a beneficial effect on animal through a combination of mechanisms that may include the reduction of inflammation and oxidative stress [23]. Several studies reported that polyphenol dietary intake increases superoxide dismutase (SOD) in cows supplemented with grape skins [24] and in growing goats supplemented with juniper oil [25]. Moreover, the durum wheat bran, fed at 10% or 20% of the supplement, due to its content in phenolic acids, especially ferulic acid, improved the oxidative status and antioxidants capacity of dairy cows and, as a consequence, of their derived cheeses [26].

However, only a few experimental studies were found in the literature concerning the response of enzymatic antioxidant defense of ruminant species to nutritional conditions occurring in farms (e.g., under and over-feeding, type and dose of supplementation) and their results are often controversial. In addition, little research attention was paid to the maintenance of redox balance in physiological conditions different from peripartum.

Since in the Mediterranean area goats kept under semi-extensive production systems could experience under-and overfeeding due to seasonal changes in forages availability, a better knowledge about the effect of nutrition on oxidative status is necessary to maintain redox balance against oxidant conditions.

In addition, to the best of our knowledge, the relationship between genotype and redox balance has received little research attention in ruminants. In goat species, the αS1-casein (CSN1S1) locus has received great attention due to its qualitative as well as quantitative implications [27]. The interaction between diet × genotype at CSN1S1 locus affects the efficiency of transformation of diet into milk and casein yield [28,29,30] and the enzymatic apparatus involved in the synthesis of medium chain fatty acids [30,31,32].

Taking into account the above consideration, in order to deepen the effect of nutrition on oxidative status parameters and to evaluate the potential effect of genotype on it, the aim of this study was to compare the oxidative response of late lactation goats characterized by different αS1-casein genotypes to different forages administered ad libitum.

## 2. Materials and Methods

### 2.1. Animals and Experimental Design

Sixty Girgentana goats from two different farms, previously genotyped at the CSN1S1, CSN1S2 and CSN2 loci, were selected. Forty multiparous goats, averaging 120 ± 10 days in milk, 1.1 ± 0.4 kg/day of milk production and 35.9 ± 6.8 kg of body weight were chosen for having the same genotype at CSN1S2 (AA) and CSN2 (AA) loci. On the basis of their genotype at CSN1S1 locus, 20 goats homozygous for strong (A/A) alleles and 20 goats heterozygous (A/F) were selected. For genotyping, hair bulbs were used to obtain goat DNA samples [33]. All samples were genotyped: (a) at the CSN1S1 locus by means of: Allele Specific-PCR (forward primer: 5′-AACGTGCCCCAGCTG-3′, reverse primer: 5′-CCT CTCCTTTAAACTTTCCC-3′, reverse primer: 5′-CTCAGCACTTTTGGGAACAAT-3′) [34]; PCR (forward primer: 5′-CATGTCAAACCATTCTATCCAAA-3′, reverse primer: 5′-CCAGCGTGATACTACTGGAAT-3′) [35]; and XmnI PCR-Restriction Fragment Length Polymorphism (forward primer: 5′-TTCTAAAAGTCTCAGAGGCAG-3′, reverse primer: 5′-GGGTTGATAGCCTTGTATGT-3′) [36]; (b) at the CSN1S2 locus by means of: NcoI PCR-RFLP (forward primer: 5′-GACACATAGAGAAGATTC-3′, reverse primer: 5′-CGTTGGGACATTTTATCT-3′) and Alw26I PCR-RFLP (forward primer: 5′-TCTCTTGCCATCAAAACA-3′, reverse primer: 5′-TGGTCTTTATTCCTCTC-3′) [37]; (c) and at the CSN2 locus by means of AS-PCR (forward primer: 5′-CGTGCTGTCCCTTTCTT-3′, forward primer: 5′-CCTCTCCTTTAAACTTTCCC-3′, reverse primer: 5′-GTTTTCCAGCTTATTCTATTTAT-3′) [38].

The goats were used in a 2 × 4 factorial arrangement of treatments, with the two genotypes (AA, AF) and four diets (DAF100; DAF65, FSF100, FSF65). Two diets included dry forage and two fresh forage. The ingredients of four diets, as percentage of fresh weight, were as follows: DAF100 pelleted diet contained alfalfa hay (98%) and vitamin-mineral premix (2%), DAF65 pelleted diet contained alfalfa hay (65%), maize (16%), barley (8%), soybean meal (3%), carob pulp (3%), corn gluten meal (3%) and vitamin-mineral premix (2%), FSF100 diet contained Sulla fresh forage (100%) and FSF65 diet contained Sulla fresh forage and barley meals (35%). The chemical composition of diets is reported in Table 1. The experiment consisted of 14 days for diet adaptation and 7 days for data and sample collection, during which the DAF100, DAF65 and FSF100 groups received the scheduled diet ad libitum, the FSF65 group received Sulla fresh forage ad libitum plus barley meal (800 g/d) divided in two meals. The dry pellet (6 mm diameter) diets (DAF100 and DAF65) have been supplied daily ad libitum. The fresh forages for FSF100 and FSF65 groups were cut daily in the morning and supplied to goats two times a day in the fodder-trough. Dry matter intake of each group was measured daily on the basis of refusals. The average intake was 2.52, 2.54, 1.82 and 1.81 kg/d for the DAF100; DAF65, FSF100 and FSF65 group, respectively. All animals were housed in individual pens where goats had free access to water and salt blocks. Animal and feeding management was accomplished by skilled personnel reflecting the common practices of commercial goat farms. On this basis, the animals were managed and housed according to the Directive 98/58/EC, as required by the Directive 2010/63/EU regarding of the protection of animals used for scientific purposes.

### 2.2. Sample Collection and Assay

Blood samples were collected at the end of the pre-experimental and experimental period from the jugular vein of each goat into two Vacutainer collection tubes. The Li-heparin containing tube was kept on ice and in the dark until their arrival at the lab and subsequently centrifuged at 1400× *g* at 4 °C for 10 min to separate plasma from the cells. The tube without anticoagulant was kept at room temperature until clotting occurred and then centrifuged at 1400× *g* for 10 min to get the serum. Erythrocytes were obtained after centrifugation of heparinized blood sample at 1400× *g* for 10 min at 4 °C and washed four times with 3 mL of 0.9% NaCl solution. The washed centrifuged erythrocytes were lysed by hypotonic shock using 2 mL of redistilled water, mixed and left to stand at +4 °C for 15 min. The aliquots of plasma, serum, heparinized whole blood and erythrocytes were stored at −80 °C for later analysis.

ROMs were measured in serum by a colorimetric assay kit (d-ROMs test, Diacron International S.a.S., Grosseto, Italy). This test measures the concentration of hydroperoxides such as hydrogen peroxide, generated by the oxidation of glucosides, lipids, amino acids, peptides, proteins and nucleotides. In the d-ROMs test, hydroperoxide groups are attacked by the iron released from plasma proteins by an acidic buffer and generate alkoxyl and peroxyl radicals. These free radicals in turn oxidize an alkyl-7 substituted aromatic amine (N,N-dietylparaphenylendiamine), producing a pink-colored derivative that can be photometrically quantified at 505 nm [40,41]. The results are expressed by using arbitrary units (Carratelli Unit, UCarr), where 1 U.Carr corresponds to 0.08 mg/dL of hydrogen peroxide.

The biological antioxidant potential kit (BAP test, Diacron International S.a.S., Grosseto, Italy) was used to measure the antioxidants by means of colorimetric assay. This test enables the measurement of many antioxidants such as uric acid, ascorbic acid, proteins, α-tocopherol and bilirubin. In the BAP test, the addition of the plasma sample to a colored solution, obtained by mixing a ferric chloride solution with a thiocyanate derivative solution, causes a decoloration, the intensity of which can be measured photometrically at 505 nm and is proportional to the ability of the plasma to reduce the ferric ions (Fe^3+^) to ferrous (Fe^2+^) iron [42]. The results are expressed in micromol/L of reduced iron.

The oxidative stress index (OSI = ROMs / BAP × 100) [43,44] was calculated in order to evaluate the relationship between oxidants and antioxidants; this relationship is often not highlighted with the determination of both components separately.

The RANSOD kit (RANSOD test, Randox Laboratories Ltd., Crumlin, Co. Antrim, UK) was used to measure SOD activity by colorimetric assay. The role of SOD is to accelerate the dismutation of the toxic radical (O^−2^), produced during oxidative energy processes, to hydrogen peroxide and molecular oxygen. This method employs xanthine and xanthine oxidase (XOD) to generate superoxide radicals which react with 2-(4-iodophenyl)-3-(4-nitrophenol)-5-phenyltetrazolium chloride (I.N.T.) to form a red formazan dye. SOD activity is then measured by the degree of inhibition of this reaction. One unit of SOD is that which causes a 50% inhibition of the rate of reduction of INT under the conditions of the assay. The results are expressed in U/mL of whole blood.

The RANSEL kit (RANSEL test, Randox Laboratories Ltd. Crumlin, Co. Antrim, UK) was used to measure GPX activity by UV assay. This method is based on that of Paglia and Valentine [45]. Glutathione peroxidase catalyzes the oxidation of glutathione by cumene hydroperoxide. In presence of glutathione reductase and NADPH the oxidized glutathione is immediately converted to the reduced form with a concomitant oxidation of NADPH to NAD+. The decrease in absorbance at 340 nm is measured. The results are expressed in U/L of whole blood.

The non-esterified fatty acids (NEFA) and beta-hydroxybutyrate (BHBA) concentration were analyzed using FA 115 and Ranbut commercial kits, respectively (Randox Laboratories Ltd., Crumlin, Co. Antrim, UK), following the manufacturer’s instructions. Glucose, cholesterol and albumin were measured by automated analyzer TARGA model 2000 (Technology Advanced Random Generation Analyzer, Biotecnica Instruments, Roma, Italy) according to enzymatic and colorimetric standard methods [46,47,48].

Samples of diets were collected at fodder-trough and they were analyzed to measure dry matter, crude protein (Nx6.25), ash and ether extract using AOAC methods [49], neutral detergent fiber (NDF), acid detergent fiber (ADF) and acid detergent lignin (ADL) according to Van Soest methods [50]. Condensed tannin (CT) content, expressed as delphinidin equivalents [51], was measured in the freeze-dried samples of Sulla and Alfalfa forage using the butanol-HCl method [52].

### 2.3. Statistical Analysis

The statistical analysis of data was performed by the analysis of variance (ANOVA) procedure of SYSTAT 13 (Systat, Software Inc., Chicago, IL, USA). The analysis included the main effect of CSN1S1 genotype (AA, AF), diet (DAF100, DAF65, FSF100, FSF65) and interaction genotype × diet. Normal distribution of the variables was assessed, and the data were ln-transformed when necessary. Data are presented as least squares means (LSM) with the standard error of the mean (SEM) in tables. Comparison between dietary treatments was evaluated with Fisher’s Least-Significant-Difference Test. Differences were considered significant at *p* < 0.05, statistical tendencies were assumed at 0.05 < *p* ≤ 0.10. Pearson correlation coefficients were calculated between the variables.

## 3. Results

### 3.1. Redox Balance

Plasma concentrations of ROMs (*p* < 0.001) and BAP (*p* < 0.05) were affected by diet; however, neither the effect of genotype nor the interaction diet × genotype was statistically significant for these parameters (*p* > 0.10). A decrease of ROMs concentration was detected in the diets with fresh forage (FSF100, FSF65) compared to the dry diets (DAF100, DAF65) (Table 2). BAP concentration was higher in FSF100, FSF65 and DAF65 groups compared to DAF100 group. The OSI index was significantly affected by diet (*p* < 0.001), but no effect of genotype and interaction diet × genotype was noted. Goats fed fresh forage (FSF100, FSF65) showed significantly (*p* < 0.01) lower values of OSI index than groups received dry diets (DAF100, DAF65) (Table 2).

### 3.2. Enzymatic Activity

**Figure 1:**
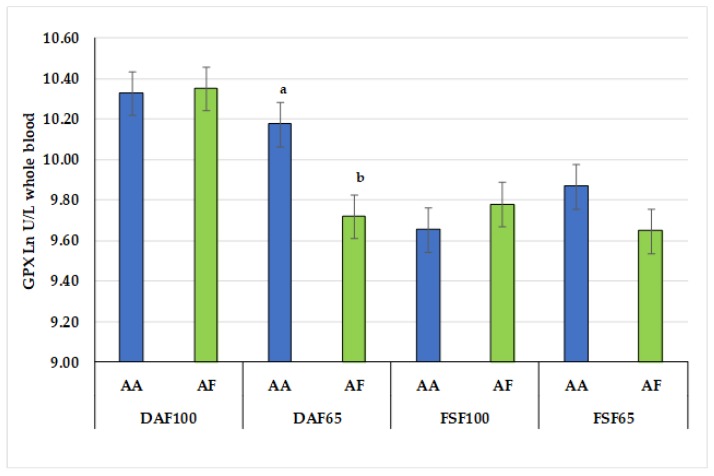
Interaction between genotype and diet for endogenous enzymatic antioxidant (GPX). Values within diets with different superscript letters are significantly different (*p* < 0.05).

Diet effect was detected on GPX (*p* < 0.01) and SOD (*p* < 0.04) activity. DAF100 group displayed the highest GPX activity, while other groups exhibited the highest SOD activity A slight influence of genotype was found on GPX activity (*p* = 0.104); in addition, a significant effect of interaction diet × genotype was observed on GPX activity (*p* < 0.036) (Figure 1) but not for SOD activity (Table 2).

ROMs concentration showed a slight negative correlation with SOD (r = −0.303; *p* < 0.086) (Table 3), while it exhibited a significant positive correlation with GPX (r = 0.541; *p* < 0.001). Moreover, a slight negative correlation between SOD and GPX was found (r = −0.22; *p* = 0.10).

### 3.3. Metabolic Profile

Serum concentrations of glucose, NEFA, BHBA and cholesterol were not influenced by diet, genotype and interaction diet × genotype. Serum albumin was affected by diet (*p* < 0.001) and its concentration increased in groups with fresh forage diets (FSF100, FSF65) (Table 2). A strong negative correlation between albumin and ROMs was found (r = −0.796; *p* < 0.001).

## 4. Discussion

A strong decrease in ROMs concentrations was observed in goats fed fresh forage when compared with groups fed dry diets (Table 2). These findings were not in line with previous observations where no significant changes in ROMs level were found in mid-lactation goats fed fresh forage compared to mixed hay plus barley [53]. However, in our study ROMs values range from 71.8 U.Carr to 260.8 U.Carr, almost as much as those listed as physiological values of the human species. Specific referral range to evaluate the redox balance in ruminants is an open topic.

The BAP test used in this study provides a global measurement of non-enzymatic antioxidants, such as proteins, ascorbic acid, uric acid, α-tocopherol and bilirubin that are indicative of short-term changes in the antioxidant status at the time of sampling [42]. In our study, higher BAP values were detected when fresh forages (FSF100 and FSF65) or diet with low hay inclusion percentage (DAF65) was administered to goats. In dairy goats it has been reported that feeding regimens based on green forage improve their oxidative statuses due to the elevated antioxidant contents of green grass; indeed, spring grazing has been shown to provide benefits to animal health due to the higher α-tocopherol content compared with summer grazing [16]. Furthermore, it is noted that diets rich in polyphenols and their metabolites exert beneficial effects on animal health through a variety of mechanisms that prevent and attenuate inflammatory response and oxidative stress [22]. Indeed, several strategies, including the intake of polyphenol-rich plants or their extracts, have been proposed to modulate ruminant oxidative status. An improvement of OSI was also detected in dairy cows that, after 100 days of lactation, were supplemented with durum wheat bran, rich in phenolic acids [26]. For example, Yerba Mate supplementation, a plant with high polyphenol concentration, seems to improve the redox balance of early-lactation cows, as reflected by a reduction of OSI value [54]. The inclusion of flaxseed plus Ascophyllum nodosum, rich of antioxidants, in the diet of late-lactation ewes showed a positive antioxidant/oxidant balance, suggesting this supplementation as a possible feeding strategy to mitigate the redox imbalance caused by environmental and physiological factors [55]. In our study, Sulla forage, which constitutes 100% and 65% of FSF100 and FSF65 diet, respectively, could have played an important role in the increase of plasma antioxidant capacity. This forage legume is characterized by a moderate concentration of condensed tannins (CT; [56]). It has been demonstrated that free or protein-complexed CT are potential biological antioxidants [57,58]. Among factors affecting plant proanthocyanidin concentration, another name of CT, forage species and period of harvesting are indicated [56]. Considering differences in CT content between two experimental legume species (19.90 g DE/kg DM vs. 0.5 g DE/kg DM in Sulla and Alfalfa, respectively) and in their storage conditions (fresh vs. dry), the differences detected in BAP values could be related to differences in CT content of diets. In a previous study, a positive correlation between CT intake and plasma antioxidant capacity was found in mid-lactation goats [53].

In order to evaluate oxidants and antioxidants jointly rather than separately, the OSI offers a better indication of redox balance [43,59]. In our study, the diets with fresh forage (FSF100, FSF65) showed a positive effect on redox balance of goats in late lactation, as highlighted by the lower values of OSI detected in these groups. The low OSI values observed in FSF100 and FSF65 groups indicate a redox balance in which the antioxidant components are greater than pro-oxidants, this plasmatic condition would seem to indicate an improvement in the health and well-being of the animal.

Based on the slight negative correlation detected between SOD and GPX, it might be that SOD and GPX do not work at the same time. It is noted that the enzymatic components of the antioxidant system possess different target sites and functions within the cells. More precisely, SOD catalyzes the dismutation of superoxide radical to hydrogen peroxide, which is further metabolized to water by GPX enzyme [60]. Similarly, a recent study, which evaluated the enzymatic and non-enzymatic antioxidants content in plasma of cow at 50–70 days of lactation and fed ad libitum with Total Mixed Ration, has shown that SOD activity was correlated only with GPX and this correlation was negative [61]. The low GPX values found in goats fed with fresh forage (FSF100 and FSF65) can be related both to the lower ROMs values, confirmed by the positive correlation between GPX and ROMs (r = 0.54; *p* < 0.001), and to the higher BAP levels detected in these groups than others, although the negative correlation found between GPX and BAP is not highly significant (r = −0.280 *p* < 0.114), as expected (Table 3).

The relationships between GPX and ROMs and between GPX and BAP observed in FSF100 and FSF65 diets seem to recur in DAF100 group but not in DAF65 group. The relationships between SOD and ROMs and between SOD and BAP are less clear than those found for GPX. This observation could be explained by the fact that the endogenous enzymatic component implements two lines of defense in temporal order and that, consequently, the substrate availability and the action sequence of the enzymes involved [62] play a decisive role in the expression of the activity of the same enzymes.

Few studies concerning the activity of endogenous antioxidant enzymes in ruminants are reported in the literature and results are often controversial. SOD and GPX activities seem not sensitive to changes in nutritional status (80% vs. 140% of energy requirements) of goats fed with dry forage diets during peripartum period and experienced some degree of oxidative stress [1]. A previous study in goats has failed to show a significant effect of diet on GPX and SOD activity in goats grazing on natural pasture and fed with dry diet [16]. A recent study reports that the feeding level causes oxidative stress in both sheep and goats but that each animal species displays a different response in enzymes activities to face the oxidative stress caused by same feeding level [20]. Precisely during underfeeding, the author found lower SOD and GPX activities in sheep and higher GPX activity but lower SOD activities in goats compared to control group; during overfeeding, GPX declined in sheep but raised in goats, while SOD activities declined in goats but was not associated to changes in sheep. Furthermore, an effect of diets with foods or by-products rich in polyphenols on antioxidant enzyme activity has been highlighted in ruminants [24,63]. SOD activity was lower in cows fed with 200 g/d/head of concentrate containing grape skins compared with group receiving the same amount of tomato pomace while GPX activity was not affected by the type of by-product [24]. According to the author, it is likely that dietary polyphenols directly deal with ROS, reducing synthesis of the enzyme. In sheep fed with diets supplemented with α-tocopherol, grape skins extracts, tomato pomace and grape skins plus tomato pomace, SOD activity was up-regulated when animals received natural antioxidants compared to control and α-tocopherol groups [63]. Clarifying the mechanisms by which the organism can counterbalance the effects of stress, the author indicates that the effect of grape polyphenols results in an enhancement of SOD gene expression. Serum GPX significantly increases in male Hu sheep fed with diets supplemented with curcumin at 450 or 900 mg per sheep compared to the control group [64].

Considering the possible relationship between natural antioxidants and enzyme activities highlighted from above studies, it is likely that the presence of polyphenols in the diet administered to goats could have affected the activities of endogenous enzymes. From in vitro tests, it emerged that this wide class of plant secondary compounds seems to act differently on different enzymatic activities and that even limited differences in the structure of polyphenols can influence not only the antioxidant capacity of the single compounds but also their ability to modulate the expression of antioxidant/detoxifying enzymes [65,66]. In a recent study, a slight increase of GPX activity was observed in goats, in mid-lactation, fed with fresh legume fodder, containing polyphenol and barley meal compared to a dry diet [53].

An interaction between genotype at CSN1S1 and diet for GPX activities was reported in Figure 1. GPX activities were higher in goats carrying strong alleles homozygous for CSN1S1 (AA), which synthesize a higher percentage of milk casein compared to heterozygous for weak alleles (AF), which synthesize an intermediate level of casein. It is not possible to speculate about this result since thus far, no study has been undertaken to assess the interaction between genotype and diet on GPX antioxidant enzyme. However, further investigations on this topic are currently in progress.

The parameters of energy metabolism found in this study indicate that goats fed ad libitum and in late lactation do not experience metabolic disorders and oxidative stress. In fact, regarding NEFA, considered the most indicative parameter for metabolic disorders [61] and oxidative stress [67], all groups displayed lower values (0.183 mmol/L) compared to those indicated as limit values of negative energy balance in the literature. Indeed, NEFA concentrations of 0.20 to 0.21 mmol/L have been suggested for lactating goats at zero net energy balances [68]. A high concentration of NEFA in blood is associated with a more frequent incidence of metabolic diseases in the perinatal period and, thus, with increasing levels of oxidative stress [61]. Additionally, NEFA oxidation in the liver increased the production of reactive oxygen species, which directly caused oxidative stress [67]. Albumin, a non-enzymatic endogenous antioxidant, is exclusively synthesized by the liver, and is the main source of plasma sulfhydryl groups (SH). Albumin is a very abundant antioxidant in plasma and it is responsible for 70% of free radical-trapping activity of serum [69]. The strong negative correlation between albumin and ROMs (r = −0.796; *p* < 0.001) noted in our study underlines the important role of albumin in the antioxidant defense system. The higher values of albumin were found in goats fed with fresh Sulla forage compared to goats fed dry forage (DAF100 and DAF65). It can be hypothesized that subjects fed fresh Sulla forage ad libitum have a more effective albumin hepatic synthesis than groups fed with alfalfa dry fodder.

The existence of intermolecular bonds between serum albumin and polyphenols was reported in several studies [70,71], indicating an important contribution of this major plasma protein to the transport of polyphenols through the blood circulation and their delivery to cells and tissue. The interaction between polyphenol and albumin may extend the life of polyphenols in the blood, allowing them to exert their antioxidant activity.

## 5. Conclusions

Data obtained in this experiment indicates that the administration of fresh and dry forages affects the redox balance of goats in late lactation. Comparing with previous studies, the effect of diet on endogenous enzymatic and non-enzymatic components is more evident. A possible relationship between polyphenol compounds of forages commonly used by goats and oxidative status biomarkers seems to be present; however, this needs to be tested in specifically designed studies. No genotype effect on redox balance was found, although an interaction between genotype at CSN1S1 locus and diet for GPX activities was noted.

## Figures and Tables

**Table 1:** Chemical composition of diets.

		Diet			
		DAF100	DAF65	FSF100	FSF65
Dry matter	%	87.5	85.7	19.1	47.1
Crude protein	% DM ^1^	15.0	15.1	17.2	15.5
Ether extract	% DM	2.1	2.3	3.2	2.8
NDF ^2^	% DM	54.2	44.3	32.5	26.7
ADF ^3^	% DM	36.8	24.6	26.9	19.4
ADL ^4^	% DM	12.9	6.7	4.9	3.5
Ash	% DM	11.3	10.4	9.6	6.71
NFC ^5^	% DM	17.4	27.9	37.5	48.3
UFL ^6^	n/kg DM	0.65	0.81	0.77	0.98

^1^ DM = Dry matter. ^2^ NDF= Neutral Detergent Fiber. ^3^ ADF = Acid Detergent Fiber. ^4^ ADL = Acid Detergent Lignin. ^5^ NFC = Nonfiber Carbohydrates, NFC = 100 − (% NDF + % CP + % ether extract + % ash). ^6^ UFL = Net energy for milk production according to [39].

**Table 2:** Least squares means of ROMs (U.Carr), BAP (Ln μmol/L), SOD (Ln U/mL whole blood), GPX (Ln U/L whole blood), Glucose (mg/100 mL), NEFA (mmol/L), BHBA (mmol/L), Cholesterol (mg/100 mL) and Albumin (Ln g/L) in dairy goats with different genotype fed dry and fresh diets ad libitum.

Item	Diet (D)				SEM	Genotype (G)		SEM	*p*-Value		
	DAF100	DAF65	FSF100	FSF65		AA	AF		D	G	D × G
**ROMs**	240.34 ^A^	260.80 ^A^	75.60 ^B^	71.79 ^B^	23.12	165.85	158.42	16.41	0.001	0.750	0.762
**BAP**	7.66 ^b^	7.94 ^a^	7.96 ^a^	8.01 ^a^	0.097	7.91	7.87	0.069	0.050	0.647	0.684
**OSI**	12.01 ^A^	9.92 ^A^	2.87 ^B^	2.44 ^B^	1.413	7.29	6.33	1.003	0.001	0.502	0.720
**SOD**	5.09 ^b^	5.35 ^a^	5.47 ^a^	5.37 ^a^	0.080	5.32	5.33	0.057	0.040	0.935	0.617
**GPX**	10.34 ^a^	9.95 ^b^	9.72 ^c^	9.76 ^c^	0.073	10.01 ^$^	9.88 ^£^	0.055	0.001	0.104	0.036
**Glucose**	51.03	49.59	51.63	53.00	1.56	50.79	50.83	1.11	0.093	0.977	0.443
**NEFA**	0.163	0.133	0.262	0.176	0.053	0.164	0.203	0.037	0.397	0.471	0.685
**BHBA**	0.377	0.340	0.287	0.512	0.064	0.425	0.333	0.045	0.150	0.166	0.296
**Cholesterol**	66.72	67.96	67.04	68.29	4.52	65.30	69.71	3.21	0.994	0.338	0.866
**Albumin**	3.31 ^b^	3.32 ^b^	3.86 ^a^	3.85 ^a^	0.02	3.58	3.59	0.01	0.001	0.665	0.395

Values within a row without a common superscript letter are significantly different: (^A^, ^B^), (^a^, ^b^, ^c^) and (^$^, ^£^) indicate significance at *p* ≤ 0.01, *p* ≤ 0.05 and *p* ≤ 0.10 levels, respectively. Abbreviations: ROMs = reactive oxygen metabolites; BAP = biological antioxidant potential; OSI = oxidative stress index; GPX = glutathione peroxidase activity; SOD = superoxide dismutase activity; NEFA = non-esterified fatty acids, BHBA = D-3-Hydroxybutyrate.

**Table 3:** Pearson correlation coefficients and probabilities significance of oxidative status biomarkers in late lactation goats.

	ROMs	BAP	SOD	Albumin
**GPX**	0.541 **	−0.280 *	−0.219 *	−0.475 **
**SOD**	−0.303 *			
**Albumin**	−0.796 **			

* *p* < 0.10; ** *p* < 0.001. GPX = glutathione peroxidase; SOD = superoxide dismutase; ROMs = reactive oxygen metabolites; BAP = biological antioxidant potential.
